# Driving singing behaviour in songbirds using a multi-modal, multi-agent virtual environment

**DOI:** 10.1038/s41598-022-16456-0

**Published:** 2022-08-04

**Authors:** Leon Bonde Larsen, Iris Adam, Gordon J. Berman, John Hallam, Coen P. H. Elemans

**Affiliations:** 1 University of Southern Denmark, SDU-Biorobotics, Odense, Denmark; 2 Department of Biology, University of Southern Denmark, Odense, Denmark; 3 Department of Biology, Emory University, Atlanta, GA USA

**Keywords:** Behavioural methods, Hardware and infrastructure, Machine learning, Animal behaviour

## Abstract

Interactive biorobotics provides unique experimental potential to study the mechanisms underlying social communication but is limited by our ability to build expressive robots that exhibit the complex behaviours of birds and small mammals. An alternative to physical robots is to use virtual environments. Here, we designed and built a modular, audio-visual 2D virtual environment that allows multi-modal, multi-agent interaction to study mechanisms underlying social communication. The strength of the system is an implementation based on event processing that allows for complex computation. We tested this system in songbirds, which provide an exceptionally powerful and tractable model system to study social communication. We show that pair-bonded zebra finches (*Taeniopygia guttata*) communicating through the virtual environment exhibit normal call timing behaviour, males sing female directed song and both males and females display high-intensity courtship behaviours to their mates. These results suggest that the environment provided is sufficiently natural to elicit these behavioral responses. Furthermore, as an example of complex behavioral annotation, we developed a fully unsupervised song motif detector and used it to manipulate the virtual social environment of male zebra finches based on the number of motifs sung. Our virtual environment represents a first step in real-time automatic behaviour annotation and animal–computer interaction using higher level behaviours such as song. Our unsupervised acoustic analysis eliminates the need for annotated training data thus reducing labour investment and experimenter bias.

## Introduction

Social communication involves multiple individuals that interact in networks, typically through multi-modal signals, such as vision and sound. Deciphering the mechanisms underlying social communication requires experimental manipulation of the complex multi-modal interactions within the social network. The field of interactive biorobotics provides unique experimental possibilities by letting animals interact with robots to understand, for example, mating behaviours^1–4^, the underlying rules of shoaling behaviour^5–8^ and communication signals^9,10^. This approach is limited by our ability to build expressive robots that exhibit complex behaviours. What passes for an expressive robot is species and hypothesis dependent, but many animals will readily accept a robot as part of their social network^11–14^. Building and controlling a small expressive robot might be possible in some cases^15^ but is often not a viable solution for small model animals due to the mechanical and computational complexity involved in fully mimicking natural behaviours.

An alternative to physical robots is to use a virtual environment, such as virtual reality (VR)^16^, defined as “a real or simulated environment in which a perceiver experiences telepresence”^17^. Current VR setups used in larval zebra fish^18^, fruit flies^19^ and mice^20^ virtualise the position of the agent in the environment by providing computer-generated visual feedback. The visual stimulus is generated by measuring the real-world movements of the agent and apply the same translation to its virtual position^18–23^. To provide a sufficiently natural virtual environment to interact with and drive the behaviour of an agent, the system needs to be fast enough to analyse, compute and generate the virtual environment within the perceptual real-time of the agent. Studying social communication in a virtual environment in most cases also requires multi-modal signals, such as vision and sound and interaction between multiple agents^24^.

A potentially excellent system for studying social behaviour in a virtual environment is vocal interaction in songbirds. Zebra finches (*Taeniopygia guttata*) live in societies and form interactive networks through calls^10,25–28^. The male song is a learned complex behaviour and is part of the mating ritual where both visual and auditory cues play crucial roles in the natural behaviour^25^. To situate a zebra finch in a virtual environment requires at least sound and vision but is likely also influenced by gaze^29^ and orientation relative to other agents^30^. Previous work has shown that zebra finches interact vocally with an immobile physical decoy providing audio from a built-in speaker^10,31^ and are physically attracted to more life-like actuated zebra finch robots^15^. Furthermore, adult finches can recognize and discriminate between conspecifics from live video feeds^32,33^ and sing song to still images^34^ or live video feeds of females^34,35^. Also, juvenile males can learn song from video and audio playback of a tutor^36,37^. Finally, perturbation of virtual auditory environments can drive active error correction of song^38,39^. Taken together, these studies suggest that zebra finches allow studying social vocal behaviour in a virtual environment. However, to our best knowledge no multi-agent setup currently exists that supports multi-modal manipulation of vocal communication, and we do not know if zebra finches exhibit normal vocal behaviour when placed in such a virtual environment.

Here we designed and built a multi-agent, audio-visual environment for experimentally manipulating social communication. Our system implementation is based on events processing that allows for much more complex computation than the state-of-the-art and a step towards integrating closed-loop control and AI in studying social communication. The latency of the current system was latency 308 ms and 383 ms for audio and video, respectively. The delay may restrict some interactive behaviours, but still allows a wide range of applications. Before using the system in biological experiments, we first wanted to test if the audiovisual environment is sufficiently realistic to elicit established behavioral responses in a social context. Therefore, in this paper we quantified several well-described social vocal behaviours in zebra finches when communicating through the environment. We show that zebra finches exhibit normal call timing behaviour to their partner, that males sing female directed song and both males and females display high-intensity courtship behaviours when communicating through the virtual environment. As an example of exploiting complex computation, we developed a fully unsupervised song motif detector. We subsequently used this detector to manipulate the virtual social environment of male zebra finches making them sing directed song to a virtual mate. Although we accepted longer system latency, our results suggest that the virtual environment is sufficiently natural to elicit courtship behaviours. Our work represents a first step in using automatic behaviour annotation for animal–computer interaction during higher level behaviours such as song.

## Results

**Figure 1:**
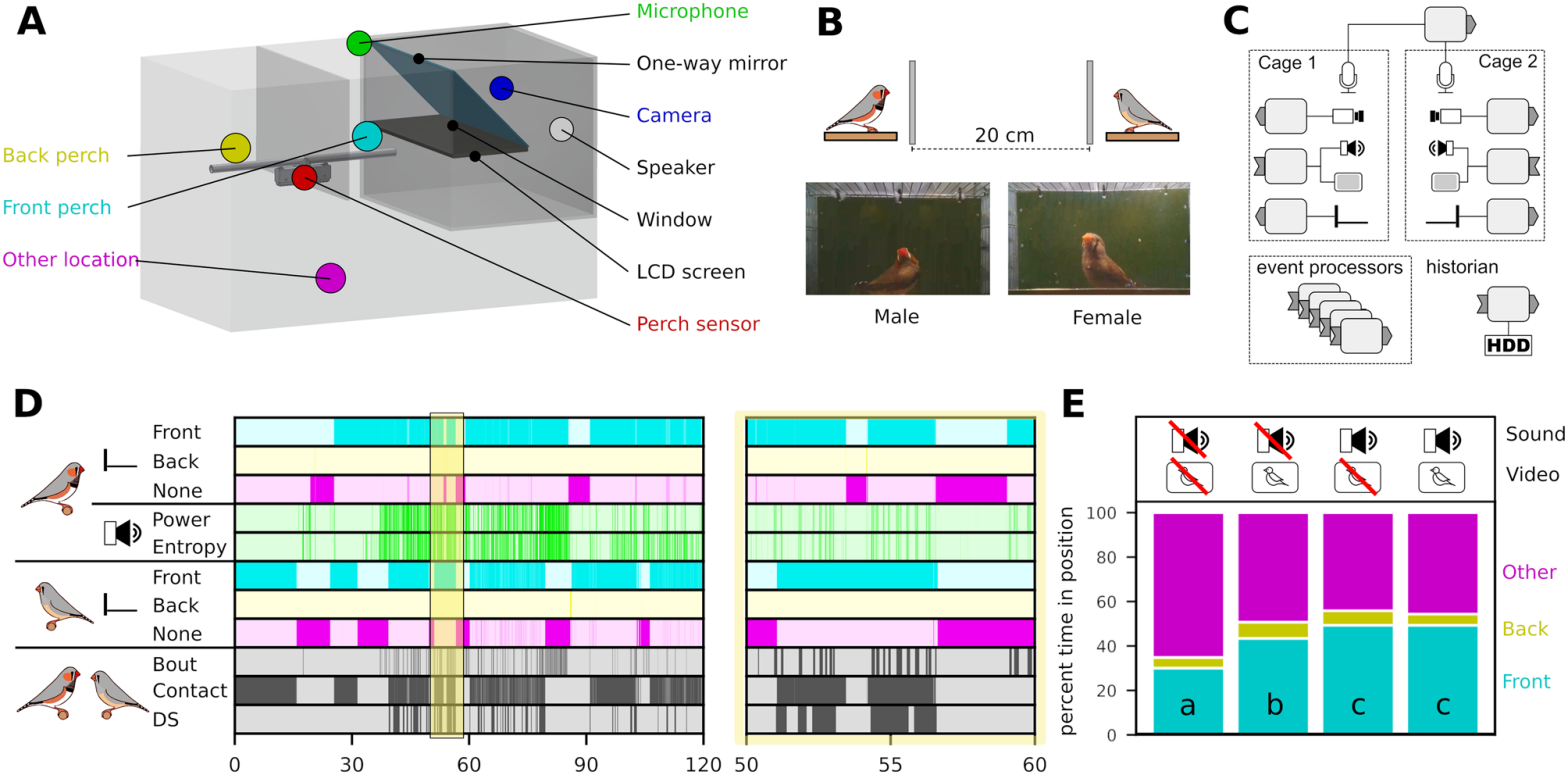
A modular multi-agent multi-modal setup for social communication in birds. (**A**) The cage equipped with the setup and an opaque divider. On the front perch the bird is able to see the screen, while not when it sits on the back perch. (**B**) Connecting two setups provides the visual impression of the other animal being twice the distance between the bird and the camera away; in this case 20 cm. (**C**) The distributed hardware architecture of the setup based on^40^. See “Methods” section for more detail. (**D**) Perch and acoustic event streams produced during 2 h of communication through the environment and a 10-min zoom of the area with yellow overlay. For complex event definitions see main text. (**E**) The perch preference in four different experimental conditions. Different letters denote significant difference (two-proportion z-test, 1% significance level, $\text {n}=24$).

We present a modular, audio-visual virtual environment able to experimentally manipulate social communication. Our system allows for multi-modal, multi-agent interaction and focuses on songbirds. Our setup is implemented in a box placed inside a cage and the cage is placed in a sound attenuating isolator box. We record and present a high-speed (60 fps) visual environment through a teleprompter system that allows direct eye contact and ensures a realistic visual perspective of the video (Fig. 1A). The cage has two perches with presence sensors (Fig. 1A); one in front of the teleprompter screen (front perch) and one behind an opaque divider that does not allow visual contact with the screen (back perch). Connecting two setups provides the visual impression that the other animal is located 20 cm away (Fig. 1B). We furthermore record the acoustic environment and present audio from a speaker located behind the teleprompter to provide the cue that sound and video have the same spatial origin. Data from all sensors is streamed on a network, translated into events using cloud-based event processing^40^ and is captured for offline processing by a node connected to storage (historian, Fig. 1C). This modular and distributed design allowed for scaling of individual parts of the system (e.g., to add sensors or software analyses) and can be extended to connect multiple setups.

Event Processing^41^ was used to represent onset and offset of behavioural features and event streams from multiple producers were combined to form new events (Fig. 1D). The position of a single bird generated three different events for absence/presence on the front or back perch or other location (Fig. 1D, cyan, yellow and magenta lines). We assumed a visual contact event when both birds perched in front of the screen. To detect and identify vocal signals, the audio stream from the male was analysed real-time (see “Methods” section) to generate events based on power and entropy threshold-crossings (Fig. 1D, green lines). A bout of song was detected by combining power and entropy with hysteresis thus suppressing most noise. Finally, a directed song (DS) event was generated when bout and contact were active at the same time (Fig. 1D, bottom line) in other words, when a male was singing while both male and female were sitting on the front perch.

To investigate the animals’ motivation for social interaction through the setup, we measured the perch preference of twelve pair-bonded male-female couples in four different audio-visual modality combinations of speaker/screen on and off. When a modality was on it was streamed to the other setup with a delay (see “Methods” section). The delays for audio and video were 308 and 383 ms, respectively. Our data showed that birds spent significantly more time on the front perch when one modality (either sound (two-proportion z-test, 1% significance level, $\text {n}=24$; $\text {z}=-6.56$, ${p}=0.0$) or video ($\text {z}=-10.10$, ${p}=0.0$)) from the other bird was on (Fig. 1E). When both video and audio modalities were on, birds also spent more time on the front perch than video-only ($\text {z}=-2.31$, ${p}=0.0105$) but not when compared to live audio ($\text {z}=1.23$, ${p}=0.8910$). This demonstrates that the birds were attracted to both audio and visual signals of another individual supplied by the system.

**Figure 2:**
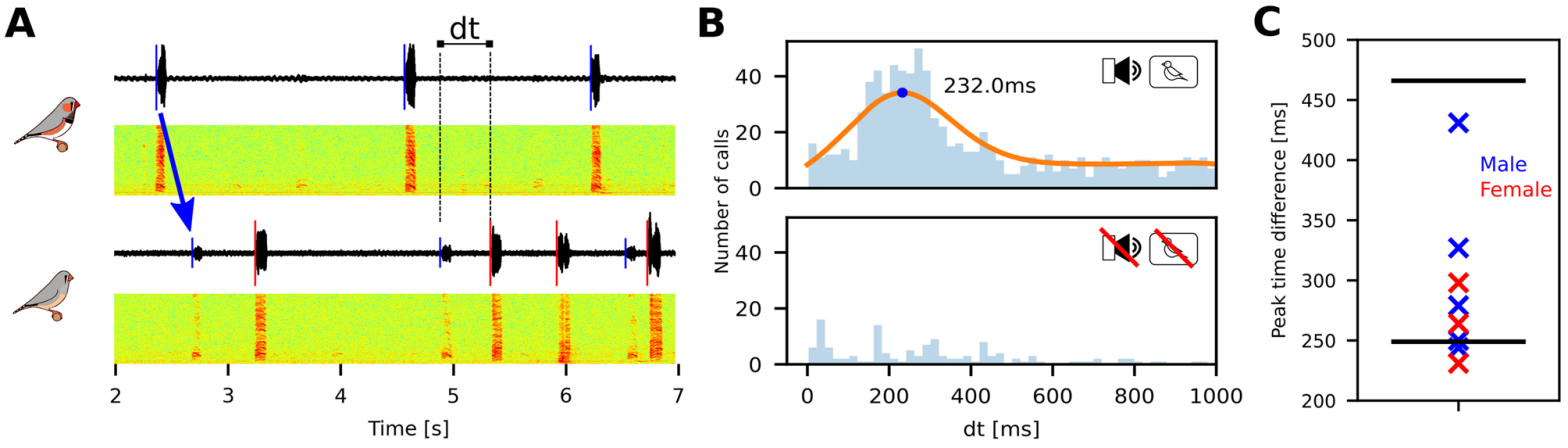
Zebra finches communicating through the setup exhibit established call timing behaviour. (**A**) Sound oscillograms and spectrograms for the stack calls of a male and a female communicating through two setups. Detected onsets are indicated by vertical lines on the oscillograms. The arrow denotes that the playback of the male call can be seen in the recording of the female. We measure the time dt from playback to answer. (**B**) Histogram of the elapsed time between the playback of the mate’s call until next call in one pair. The peak of the kernel density estimate is marked. Bottom plot shows a histogram of time differences for the same pair with the system off. (**C**) Summary showing the peak for all 8 birds. The black lines show the range of values reported in^10^.

To demonstrate that the setup provided a sufficiently natural social environment, we exploited two key behavioural responses in communication between pair-bonded individuals: call timing and directed song. Coordinated call production between partners is a well described behaviour in birds, where it is thought to influence pair-bond maintenance and mate guarding^42^. Zebra finches show time-locked call behaviour using two types of calls: Tet and stack calls^26^. Both are short, low power vocalizations used when the birds are physically close together^25,28^. We quantified the call timing of calls between established pair bonded couples communicating through our virtual environment (Fig. 2A) and identified calls using a supervised random forest classifier (see “Methods” section). With both audio and visual modalities on, the delay from hearing a call to producing one was unimodally distributed (Fig. 2B) with a peak delay at 291 ms (median: 271 ms, range: 231–431 ms, N = 8, Fig. 2B,C). This data is consistent with previous published ranges of call timing delay (249–466 ms^10^ and 68–283 ms^27^). Because calls were synchronized and contingent on the call of the mate, we conclude that the birds displayed natural call timing behaviour through the virtual environment.

Next, we studied whether males exhibited natural singing behaviour to their virtual mate. Male zebra finches sing both to females (directed song, DS) and not directed towards any particular conspecific (undirected song, US)^25^. The song consists of introductory notes and a stereotyped sequence of syllables, called the motif, that is often repeated several times to form a song bout^25,43^. Although the DS and US motif consist of the same syllable sequence, several key acoustic features are different between DS and US. The DS motif is delivered faster and is preceded by more introductory notes^44^. It also has more repetitions of the motif in each bout, increased sequence stereotopy^43^ and DS syllables exhibit less variation in the fundamental frequency (FF) of harmonic stacks^45^.

**Figure 3:**
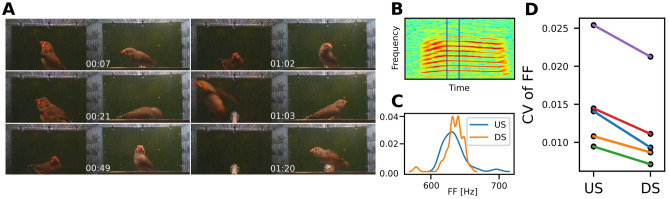
Adult male zebra finches sing directed song to their mate through the virtual environment. (**A**) Male (left) and female (right) displaying behaviours associated with directed song. (**B**) Spectrogram of a syllable with harmonic stack used to estimate fundamental frequency. The vertical lines indicate the part of the syllable used to estimate fundamental frequency. (**C**) Kernel density estimates for the fundamental frequency of the syllable above based on 30 renditions with the system on (DS) and 30 with the system off (US). (**D**) The Coefficient of Variance of the fundamental frequency is significantly lower when the virtual environment is on (Wilcoxon signed rank test 10% significance level, $\text {n}=5$, $\text {W}=15$, ${p}=0.0312$, ${\text{CLES}}=0.76$) indicating DS.

We studied five established pair-bonded couples communicating through our environment and we isolated candidate DS events as the simultaneous occurrence of bout and contact events, i.e., when both animals were perched in front of the screen and the male was vocalizing. The video segments of potential DS events were subsequently scored for accompanied behaviour by experienced observers (IA, CPHE). All (5/5) males sang directed song to their virtual mates and displayed courtship behaviours, such as fluffing, beak wipes and jumping, that are indicative of DS (Fig. 3A) at high intensity. The coefficient of variance (CV) of the fundamental frequency (Fig. 3B,C) was significantly lower (Wilcoxon signed rank test 1% significance level, $\text {n}=5$, $\text {W}=15$, ${p}=0.0312$, ${\text{CLES}}=0.76$, Fig. 3D) when the virtual environment was on compared to off further indicating DS. Taken together our data strongly suggest that all males sang DS to their virtual mates.

In summary, call timing between individuals was comparable to that of freely communicating animals and males sang DS to their virtual mates, which showed that our virtual environment provided a sufficiently natural environment for normal behavioural responses in songbirds.

A final crucial component in the design of a closed-loop system is the ability to manipulate an agent’s environment and thereby drive its behaviour within its perceptual real-time. When zebra finch males sing DS to a female, they typically habituate to its presence, which leads to a reduction in the number of motifs per minute^44^. In experiments requiring extended periods of DS and/or a high number of motifs, this effect is typically countered by introducing novel females to reinvigorate the male^44,46^. Here we aimed to drive DS behaviour by presenting different virtual females based on the measured song performance of the male.

**Figure 4:**
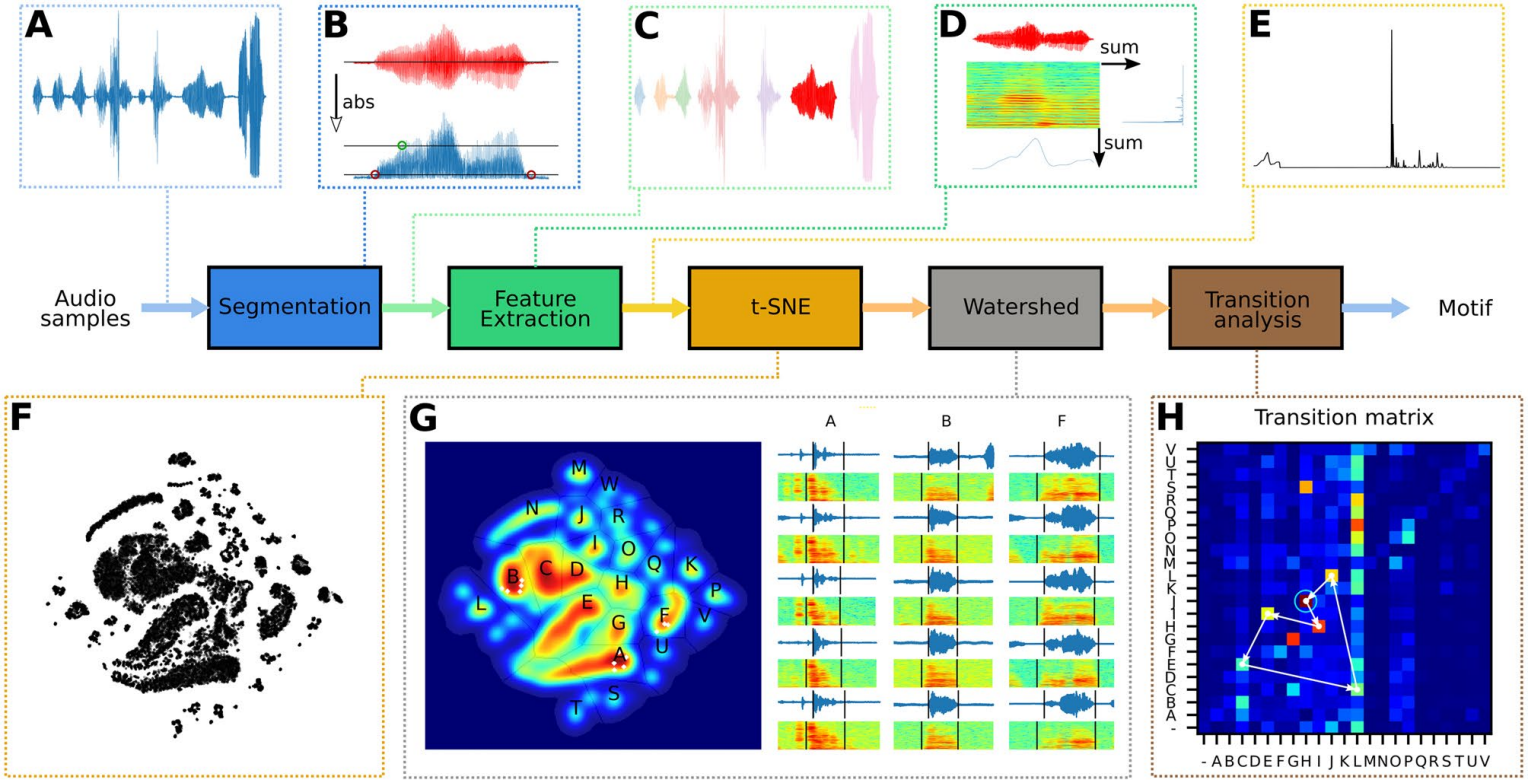
Unsupervised training of motif detector pipeline. (**A**) The raw sound signal is received as a continuous stream. (**B**) When the absolute value of the samples crosses the high threshold, we search back and forth for onset and offsets based on crossing of the low threshold. (**C**) The segmented signal. (**D**) A spectrogram of the sound is generated and two vectors are computed by summing the rows and columns, respectively. (**E**) The two vectors are normalised and concatenated to a 746-dimensional feature vector. (**F**) Dimensionality reduction of 60k feature vectors into a 2D space shows that sound segments cluster together. (**G**) Regions of stereotyped sounds are labelled. Examples of randomly picked sounds from three different regions are indicated by white dots on the density plot and with oscillograms and spectrograms on the right. Each column is a different region. (**H**) The most common song motif is extracted by forming the transition probability matrix and following the most likely transitions forming a loop. The loop represents the most stereotyped sequence and is defined as the motif.

Driving song behaviour based on song performance requires detection of the stereotyped syllable sequence, i.e., the motif. Therefore we developed a novel, unsupervised motif detector (see “Methods” section). The detector is based on dimensionality reduction of feature vectors generated from the spectrogram of sound segments (Fig. 4A–E). Training of the model was based on 60,000 feature vectors per animal, each representing a segment of sound. The feature vectors were embedded in a 2D space using t-distributed Stochastic Neighbor Embedding^47^ and the watershed transform^48^ was used to cluster the space into a behaviour map^49^. Next, we computed the transition probability matrix between all syllables in the training data (Fig. 4H) and used it to detect the most stereotyped sequence of syllables by starting at the globally most likely transition and following the path of locally most likely transitions (Fig. 4H). In all the males, this path contained a cycle that we defined as the motif of the individual and it was confirmed by experienced observers (IA, CPHE) to be the correct motif.

**Figure 5:**
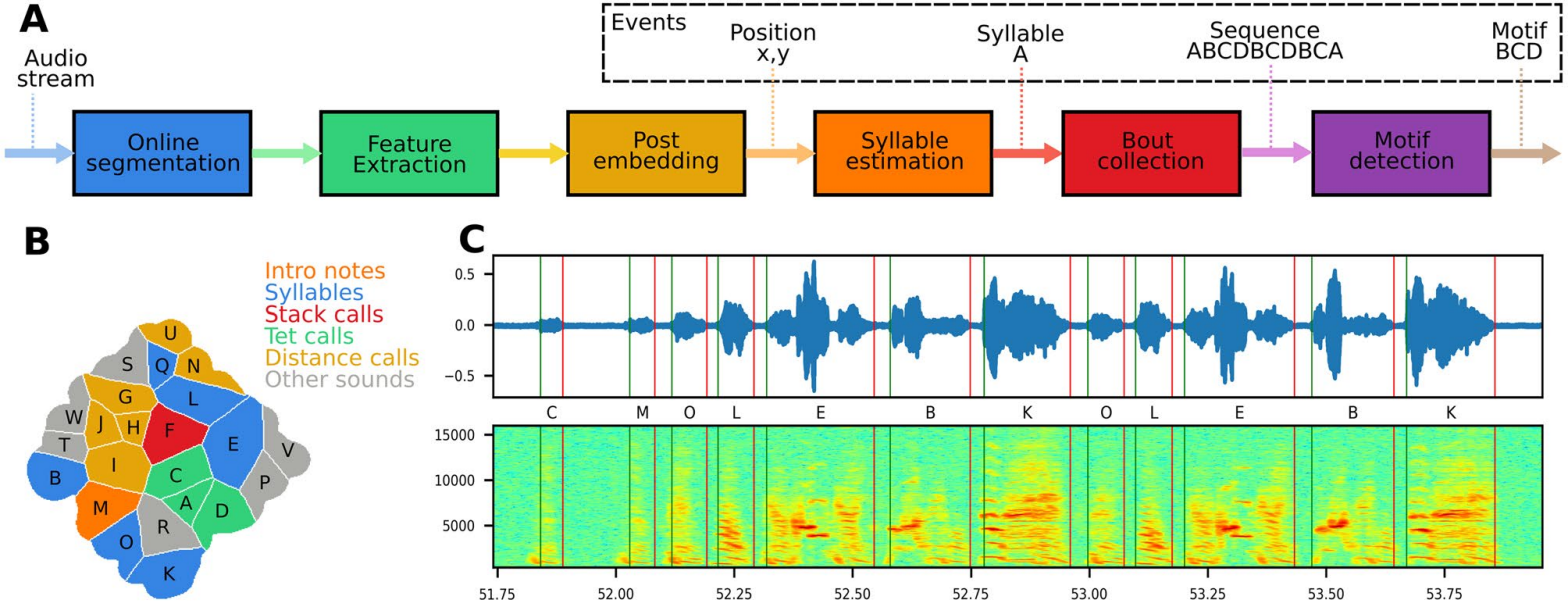
Analysis pipeline for real-time motion detection. (**A**) The pipeline for analysis uses the methods described in Fig. 4A–E for segmentation and feature extraction. The resulting feature vector is post-embedded in the 2D space and classified based on which region it falls within. Syllables are collected in sequences and screened for motifs. (**B**) The behaviour map annotated manually based on sample sound segments from each region. (**C**) Song spectrogram with vertical lines indicating onset (green) and offset (red) events of automatically detected syllables. The letters indicate the class of the syllable.

Next, we extended the method to detect motifs real-time (Fig. 5A). We detected syllable events by analysing the audio stream and post-embedding the sound segments into the previously computed 2D space (Fig. 5B). Syllable events were then collected in sequence events that were screened for ordered subsets of the motif (see “Methods” section) to create the motif event (see example in Fig. 5C). The entire process was parallelised to achieve real-time detection.

**Figure 6:**
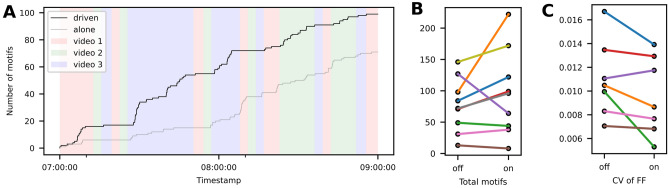
Number of directed motifs can be increased using real-time motif detection. (**A**) Cumulative sum of detected motifs for one bird during a 2-h period of driving the behaviour by switching virtual female individuals and the same 2 h on the following day without video. Background colours show which video was playing during that time. Videos change dynamically based on number of motifs. (**B**) The number of motifs sung was significantly higher (Wilcoxon signed rank test 5% significance level, $\text {n}=8$, $\text {W}=33$, ${p}=0.0195$, ${\text{CLES}}=0.625$) during the 2 h with video compared to the 2-h control without video demonstrating that the video drives them to sing more. (**C**) The coefficient of variance for the fundamental frequency showed a trend to be lower (Wilcoxon signed rank test 5% significance level, $\text {n}=7$, $\text {W}=4$, ${p}=0.0547$, ${\text{CLES}}=0.612$) during the 2 h with video compared to the 2-h control. Colored lines in (**B**) and (**C**) indicate individuals.

We exposed males to 1 min audio-visual recordings of one female in an excited state from the DS experiments, as long as motifs were detected (see “Methods” section). After 3 min without motif detection, we switched to the audio-visual recordings of another female. Driving the behaviour over 2 h, males sang significantly more motifs (Wilcoxon signed rank test 5% significance level, $\text {n}=8$, $\text {W}=33$, ${p}=0.0195$, ${\text{CLES}}=0.625$) compared to the control US period (Fig. 6A,B). Because we did not randomize the experimental sequence, we cannot exclude that treatment ordering contributed to the observed effect. To confirm that the birds sang DS motifs, we computed the CV of FF in a motif syllable containing a harmonic stack. The CV had the trend to be lower during the driving period compared to the US control period, which suggests that the males sang DS to the virtual females (Fig. 6C). Taken together, our system made the birds sing (i) directed motifs and (ii) more so in 2 h compared to undirected motifs in the control period, thus demonstrating the ability to drive directed singing behaviour.

## Discussion

We present an experimental environment to study social communication that allows multi-modal, multi-agent interaction. Our goal was to first test if the audiovisual environment was sufficiently realistic to elicit established behavioural responses in social communication, before using the system in biological experiments. Zebra finches communicating within the modular virtual environment emitted calls that were synchronized and contingent on the call of the mate with response latencies as in real life situations^10,26,27^. Furthermore, our data show that males exhibited high-intensity courtship behaviour and sang directed song to their virtual females. To detect DS events, we used an easily implemented definition of DS as song that occurs assuming visual access. Previous studies also defined DS as song when the male was singing oriented towards a conspecific female^34^, but did not confirm this classification by further acoustic analysis such as decreased DS motif duration^44^, or decreased variation in the fundamental frequency of harmonic stacks in DS syllables^45^. Both, behavioural and acoustic analysis support that song elicited under our definition was indeed DS. Taken together, these data suggest that the virtual environment was sufficiently realistic to elicit essential behavioral responses and courtship behaviours from both sexes.

Furthermore, by switching videos of excited females we could keep males to sing DS. Whether the increased amount of song was driven by video switching we cannot answer as this would require additional controls. Dissecting out the sensory and behavioral cues that trigger DS in a male and escalation of the courtship intensity are few of many of the interesting biological questions that will require complicated image and sound manipulation to answer. This would be a major advance that we hope our system will be able to contribute in the future.

We present and implemented a syllable-based unsupervised audio classifier which we think will be widely applicable in bioacoustics. Unsupervised clustering methods have been used in the analysis of vocalisations^50^ but are typically based on a few dozen acoustic features. A more data-driven approach is to use spectrograms directly as high dimensional features^51^, which however imposes extensive computational costs. Here, we compressed the spectrograms to arrive at a manageable sized feature vector thereby keeping computational costs low. Especially for stereotyped behaviours, unsupervised methods like t-SNE^47^ excel because clusters of repeated behaviours stand out from noise^49^. Furthermore, we parallelized a variation of the post-embedding algorithm described in^49^ to achieve real-time classification. Lastly, we could determine each individual’s motif in an unsupervised way by assuming only that the motif is the most repeated syllable string, thus exploiting the fact that zebra finch song is highly stereotyped. Our unsupervised method eliminates the need for annotated training data and thereby reduce labour investment and the risk for experimenter bias.

We acknowledge that our system latencies are rather large and can be optimized—though we note that the zebra finches displayed normal behavioral responses. There are three causes for these latencies. First, any modern video system copies a captured image as digital data, from the capture hardware to the render hardware, and this can only be avoided by custom design. Our choice of commodity components imposes a minimal latency because this unavoidable transfer time depends on the bus speed (and therefore the cost) of the computers used. It is always at least 1 frame period (17 ms). Second, given our architectural decisions to decouple and modularize, the data had to be transferred via network which again imposed a (cost-related) latency. The raw data rate of the camera is about 74 Mbit/s, necessitating gigabit networking and a delay of around 10 ms to transmit a frame. Further, jitter in the network travel time requires a jitter buffer at the rendering system to ensure uninterrupted timely presentation of frames. In all, this implies a minimal latency of about 60–80 ms for our system. The system latency can be reduced with faster, more expensive components.

Our setup represents a first step in closed-loop automatic behaviour annotation and animal–computer interaction using higher level behaviours. We used events to represent behaviour and event-processing and microservices to achieve near real-time capabilities^40^. Several studies have demonstrated the power of near real-time processing in closed loop assays in neuroscience^52,53^, to manipulate pitch in songbirds^54–56^ or to provide virtual reality^18–20^. Those studies take advantage of computationally attractive features, such as action potentials or acoustic features, to allow near real-time system response. We acknowledge that the implementation of our system architecture is slower, but we chose to devote engineering effort and resources to automating analysis of higher levels of behavioural organisation - which required a different architecture and higher computational load than previous works - rather than latency optimization. One interesting future application of our system would be to systematically alter latency between modality stream to study the tolerance for delay on the dynamics of vocal exchanges.

Our system is modular and can be extended to multiple setups or to add more sensors, actuators, and computational units. We expect our modular setup to be applicable to other species of social birds and mammals. For example the sample rate of the audio interface can easily be increased to allow ultrasonic vocal communication in rodents. We deliberately based the setup on cheap distributed computers, free and open-source software, and cloud computing to ease the reuse of hardware and software modules in other projects, make it easier for multiple developers to contribute, and make the implementation more widely accessible. The distributed architecture complicates the system and increases the minimum latency, but allows it to scale linearly, makes it easier to maintain, and makes it resilient to single node failures. The system can be deployed anywhere with network and thereby enables global-scale social communication experiments. Setups situated around the globe could thus be connected and allow for unique long-term communication experiments between labs that are physically far apart.

## Methods

### The virtual environment

The virtual environment was built on the teleprompter principle, where a slanted one-way mirror allows the camera to record the bird through the mirror while the bird sees the reflection of a screen below (Fig. 1A).

A microphone is placed outside the cage above the perch in front of the screen while the rest of the system is placed inside a painted wooden box placed in the cage. The one-way mirror is constructed from a sheet of 3 mm transparent acrylic plexiglass coated by 0.02 mm silver one-way film with 70% light admittance and 99% reflectance.

The screens are trichromatic 7″ LCD displays in 800x480 pixel resolution. Although birds possess at least tetrachromatic or even pentachromatic vision^57^, previous studies showed that males sing when presented with live video of conspecific females on trichromatic screens^32,34,35^. However, critical to eliciting courtship behaviour was the use of 100 Hz screens^32,35^ that are above the flicker-frequency of birds^57,58^ or non-flickering liquid-crystal displays (LCD). Therefore, we decided to use 60 Hz LCD screens that present slower, but continuous, flicker-free images to the birds. The video is recorded with a Raspberry Pi Camera V2 in 800x480 pixel resolution at 60 frames per second (fps) and streamed to the network from a Raspberry Pi 3. The video delay was measured by simultaneously turning on an LED in both boxes and recording a video with an external camera showing both the LED and the screen. By counting the number of frames from the local LED turns on to the remote LED from the other box can be seen on the screen the delay can be calculated. This delay was measured to 383 ms (23 frames at 60 fps).

The audio playback comes from a 1.5 W mini-speaker placed behind the mirror. While originally playing sound at the received level of the microphone in the other box, we slightly attenuated this signal (-6dB) to avoid acoustic feedback. Because a 6dB attenuation corresponds to a doubling of distance of the source^59^, the bird in box would perceive the source level of the animal in box 2 as if it were two times further away than it was. However, because the source level of zebra finches in the lab varies from 50–70 dB reference $20 \,\upmu {\text{Pa}}$ at 1 m^60^, a 6 dB attenuation is well within the natural range of variation. Since we form a loop between the two cages a bird could hear a delayed and strongly attenuated version of its own vocalisation similar to hearing an echo. We measured the total attenuation to vary between 15 and 30 dB.

The sound was recorded and streamed from a multi-channel recording array^61^ using Knowles FG23329-PO7 microphones. The recording equipment is not part of the developed virtual environment and it could be replaced by any system capable of streaming audio. The audio delay was measured by making a loud sound (with a clicker) in one box and timing the difference between that signal in one box and the version played back in the other box. This delay was measured to be 308 ms ± 4 ms. The audio to video leading delay was thus 75 ms. This delay is about two times below the $180\pm 42 \,{\text{ms}}$ delay to perceive lip-syncing errors in humans^62^, but we are not aware of any comparative data in birds.

Figure 1C shows the architecture of the system. Each module contains two Raspberry Pi 3 model B connected to a gigabit switch. One is connected to the camera and is only responsible for streaming video. The other, connected to display and speaker, is responsible for playback of sound and image. A third Raspberry Pi 3 is placed on top of the cage responsible for polling the perch sensor at 20 Hz and emitting state changes as events. It also measures temperature and humidity in the box and emits those as events every minute. The multi-channel microphone array is placed outside the isolator box with a microphone placed in each cage. All computers on the network are synchronised to within milliseconds using the Network Time Protocol^63^ implemented with chrony^64^.

Data is streamed to IPv6 multi-cast groups following the publish-subscribe pattern^65^. A PC in the bird room acts as historian, saving the data streams. Data is offloaded to a Ceph^66^ persistent storage cluster placed in our data centre. Several event processors continuously analyse the data streams, producing events. These are running in a docker swarm^67^ cluster also in our data centre.

The two-layered architecture is based on data streams and event streams^40^. An event is an association between a specific time and a specific property, in this case a behaviour. A data stream contains sampled data from sensors such as cameras and microphones while an event stream consists of events produced by data stream processors or by asynchronous sensors such as contacts.

The continuous audio stream is analysed real-time to produce the power and entropy events. This analysis is based on estimating the power by squaring the sample values and the entropy as the ratio of the geometric mean to the arithmetic mean. The analysis is implemented as plug-ins to the media-streaming framework gstreamer^68^. The estimates are thresholded with hysteresis^40^ and published as kafka events^69^. Hysteresis was used to prevent so-called switch bouncing between on and off when the input signal was close to the noise level^70^. A perch sensor installed in the cage directly generates a perch event every time the bird changes location in the cage. Based on those three events, three complex events are generated, namely bout, contact and directed song. The bout event is active when both power and entropy events are active, and the contact event is active when both birds are perched in front of the screen. The directed song event is active when the bout and contact events are active (Fig. 1D). Event processing was implemented as microservices in docker containers^67^ for high modularity and was running in our data centre.

### Animals and husbandry

Adult male and female zebra finches (*Taeniopygia guttata*) were kept pairwise in breeding cages at the University of Southern Denmark, Odense, Denmark on a 12 h light:dark photoperiod and given water and food ad libitum. All experiments were conducted in accordance with the Danish law concerning animal experiments and protocols were approved by the Danish national ethics committee, the Danish Animal Experiments Inspectorate (Copenhagen, Denmark). The reporting in the manuscript follows the recommendations in the ARRIVE guidelines.

We used adult zebra finches ($> 100\,{\text{days}}$ post hatch) that were established breeding pairs (meaning that they had produced at least one clutch of offspring together) in the animal-animal communication experiments and additionally also single males for the animal–computer experiments. All animals were naive to the experimental conditions. When not in experiment, the birds were kept pairwise in breeding cages or in aviaries containing 200-300 individuals. Under experiment the birds were isolated in sound-attenuated boxes for a maximum of ten days before returning to their usual surroundings. The birds had access to food and fresh water ad libitum served at the bottom of the cage and from feeders at the side of the cage. In the virtual environment, the birds were kept on a 12 h light:dark photoperiod. The temperature was kept between 22 and 28 $^{\circ }$C and the relative humidity at 50–60%. The temperature difference between the position in front of the screen (front perch) and behind the blind (back perch) was measured with the system fully on to be 0.4 $^{\circ }C$ ($\pm 0.3\%$ accuracy). The isolator boxes attenuated sounds in the 200–8000 Hz range by 40dB (measured by playing back sound in the isolator and record sound levels both inside and outside the box. A fan ensured air flow in the box and provided cooling for the equipment located inside.

### Sound segmentation

Segmentation was based on the silence between syllables and was calculated from the amplitude of the signal (Fig. 4B). We used two threshold values applied on two different metrics for discriminating between sound and silence. The input signal (Fig. 4A) was normalised to range [−1;1] but otherwise not pre-processed. The high threshold was 0.5 and the metric that had to pass this threshold was the absolute value of the signal. The low threshold was also 0.5 but the metric having to go below this threshold was the peak-to-peak amplitude over 325 samples. Both threshold values were determined experimentally based on initial recordings. Starting from the $i_{on}$th sample where the absolute value of the sample $s_i$ surpasses the on-threshold $t_{on}$ (0.5)

1 $$\begin{aligned} i_{on} : \left| s_i \right| > t_{on} \end{aligned}$$

we searched backwards in time to find the onset sample number $i_{onset}$ defined as the sample where the peak-to-peak amplitude over *w* samples (325) was below the off-threshold $t_{off}$ (0.5).

2 $$\begin{aligned} i_{onset}: max(s_{i-w}:s_i) - min(s_{i-w}:s_i) < t_{off} \end{aligned}$$

Similarly we searched for the offset sample number $i_{offset}$ as new samples arrived.

3 $$\begin{aligned} i_{offset} : max(s_{i}:s_{i+w}) - min(s_i:s_{i+w}) < t_{off} \end{aligned}$$

As soon as the last sample was received the segment was passed on to the next stage of the pipeline. Segments shorter than 30 ms or longer than 300 ms were discarded since the duration of zebra finch syllables is expected to be within that range. We implemented both an online and offline version of this segmentation algorithm and used it for all the experiments presented in this paper.

### Perch preference protocol

The same data was used for all the animal-animal communication experiments. The virtual environment was powered down for at least 2 h before the birds were moved to the isolator box (day 0) and left off for at least 24 h before it was turned on for another full day (day 1). Experiments ran on the following days starting when the cage lights were turned on and for 2 h thereafter. Day 2 was always with black screen and no sound and the following days the system cycled through perturbations of two speaker states (off, on) and two screen states (off, on) in randomised order. After the experiments the birds were returned to their home cages. To investigate the motivation for using the virtual environment, we looked at the perch preference in different states of the system. Based on the perch sensor, an event was emitted every time the bird changed position in the cage and summing the duration of the events gives a measure of the proportion of time spent in each position (Fig. 1E).

### Call-timing protocol

The audio was segmented as described above and combined into one big dataset covering 12 h a day for all 12 pairs. To provide training data, we then hand-annotated for each bird the first 30 min with both video and audio on. To ease annotation, we used pre-clustering based on cross-correlation, so the observer was presented with oscillograms, spectrograms and sound from 1 min at a time that had already been clustered into groups of sounds with high cross-correlation maximum. To prevent false negatives we used very low threshold to ensure we detected all sounds. The observer then had to name the groups and correct mistakes made by the pre-clustering algorithm. The classes found were song, “stack” calls, distance calls, echo (loud sounds from the other bird triggering the segmentation), wing flapping and noise. Classification of vocalisations followed the descriptions in^25^. We did not observe “tet” calls. Based on the annotations a random forest classifier^71^ with 100 estimators was trained for each bird ranging in mean accuracy (10% hold out) from 0.82 to 0.96. To investigate call timing, we measured the time difference from the playback of a stack call (onset + delay) to the next stack call emitted by the animal of interest up to a maximum of 2 s. Histogram of the time differences were constructed (200 bins) and plotted with Gaussian Kernel Density Estimates (KDE, bandwidth=100, Fig. 2B).

### Directed song protocol

To confirm directed song in the animal-animal communication experiments, we selected videos with potential female directed song based on the definition that both birds were on the front perch and the male was vocalising. The videos were then scored by experienced observers (IA, CPHE) for the display of hopping, jumping, beak wiping, looking at the mate and fluffing plumage (see Fig. 3D for examples). As a quantitative measure, we calculated the coefficient of variance of the fundamental frequency, which is lower in DS compared to US^45^. However, this measure is extracted from stack syllables without frequency modulation such as the one shown in Fig. 3B. The motif of five birds contained a suitable syllable. From spectrograms of the motifs identified by the motif detector, we manually selected the same place in the stack syllable (Fig. 3B) in 30 motifs from each individual. The fundamental frequency (FF) was estimated from 2048 ($\tilde{4}2$ ms) samples using the YIN algorithm^72^. Because the reduction of CV of FF during directed song has only been established during harmonic stacks^45^, one male that did not have a harmonic stack in its motif was excluded from this analysis.

### Animal–computer communication experiments

For the driving experiments, the male was left to habituate to the new surroundings until he produced at least 10 motif repetitions during the first 2 h after lights on (day 0). On the following day (day 1) we ran the driving experiment, meaning that videos were displayed showing excited females. Three videos of different females were used, each 1 min long, taken from the animal-animal communication experiments. In case of pair-bonded males, the established mates of the focal animals were not among the female videos. In case of single males the 60,000 training samples were recorded over three days prior to the experiment. The logic of the system is that every time a motif is detected, a timer is reset. If the timer ran out (3 min since last motif) the next video was displayed and otherwise the same video kept getting looped. On day 2 we recorded the control without video playbacks.

### Motif detector

We trained one motif detector per individual. The feature extraction is based on summing the rows and columns of the spectrogram (Fig. 4D) and concatenating them to form a feature vector (Fig. 4E).

First a spectrogram of the segment is formed by applying Short-Time Fourier Transform (STFT) with FFT size of 1440 and stride of 25 samples. The parameters are all based on the sampling frequency of 48 kHz, the duration of sounds (30–300 ms) and the desired number of time bins. The smallest spectrogram we can make has just one time bin and thus the maximum FFT size is:

4 $$\begin{aligned} FFT = 30\,{\text{ms}} * 48\,{\text{kHz}} = 1440\,{\text{bins}} \end{aligned}$$

The stride parameter can then be calculated:

5 $$\begin{aligned} stride = ((300\,{\text{ms}}*48\,{\text{kHz}})-1440\,{\text{bins}})/512\,{\text{bins}}) = 25.3125 \approx 25 \end{aligned}$$

The spectrogram is cropped to the approximate audible range for zebra finches 200 Hz to 8 kHz (234 bins) and the time dimension is cropped to the first 512 time bins corresponding to 300 ms (zero-padded if the segment duration is shorter). The rows and columns of the spectrogram are summed and the two resulting vectors $F_t$ and $F_f$ are concatenated to form a 746-dimensional feature vector *F* (Fig. 4E).

6 $$\begin{aligned} F_t= & {} \sum _{t} STFT(t,f)\end{aligned}$$

7 $$\begin{aligned} F_f= & {} \sum _{f} STFT(t,f) \end{aligned}$$

8 $$\begin{aligned} F= & {} \left[ F_t \ \ F_f \right] \end{aligned}$$

Each vector is normalised to have a sum of one before concatenation.

A training set is created consisting of 60,000 feature vectors from the same individual, each representing one sound segment. We embed each of these high-dimensional points in a two-dimensional space (Fig. 4F) using the t-SNE method introduced in^47^. The method minimises the relative entropy between two distributions, one representing the high-dimensional points and one representing the low-dimensional points, so that close points in the high-dimensional space are also close in the low dimensional space.

Since we are interested in stereotyped behaviour, we then followed the method described in Berman et al.^49^ placing a Gaussian kernel (bandwidth = 15) on each embedded point. We then generated a density plot (Fig. 4G), and we detected all peaks that are separated by a distance of 15 or more. Using the watershed algorithm^48^ on the inverted density plot, we get a set of clusters, each representing roughly a stereotyped syllable. Examples from three different regions can be seen in Fig. 4G. The further a point is from the peak of the region the more likely it is to be mis-classified and thus distance from peak could be used to indicate certainty of the classification. We found that some regions represent a merge of two syllables while some represent part of a split syllable. For higher accuracy in detecting the syllables this information could be used for post processing or better means of segmentation could be introduced.

After the training phase a new data point *z* is embedded based on the already embedded points, largely using the method described in^49^ appendix D. The perplexity parameter of the t-SNE algorithm can be interpreted as a measure of the number of nearest neighbours^47^ and therefore we only consider the ’perplexity’ nearest points *X* in the high dimensional space found using the ball tree algorithm^73^.

We then choose an embedding $z'$ of the new point *z* such that conditional probabilities in the low-dimensional space $q_{j|z'}$ are similar to those in the high-dimensional space $p_{j|z}$. The conditional probability of a point $x_j \in X$ given the new point *z* is:

9 $$\begin{aligned} p_{x_j|z} = \frac{D_{KL}(z||x_j)^2 / 2\sigma _{z}^2)}{\sum _{x\in X} D_{KL}(z||x)^2/2\sigma _{z}^2)} \end{aligned}$$

where *X* is the vector of nearest points in the high-dimensional space, *z* is the new point in the high-dimensional space, sigma is found by a binary search for the value that produces a conditional probability with the perplexity set by the user and $D_{KL}$ is the relative entropy given by:

10 $$\begin{aligned} D_{KL}(P||Q) = \sum _{x \in X} P(x)log \left( \frac{P(x)}{Q(x)} \right) = - \sum _{x \in X} P(x)log \left( \frac{Q(x)}{P(x)} \right) \end{aligned}$$

The conditional probability of a point $x'_j$ in the low-dimensional embedding $X'$ given the new embedding $z'$:

11 $$\begin{aligned} q_{j|z'} = \frac{(1+\Delta _{j,z'}^2)^{-1}}{ \sum _{x' \in X'} (1+\Delta _{x',z'}^2)^{-1}} \end{aligned}$$

where $\Delta _{a,b}$ is the euclidean distance between the points *a* and *b*. Since $z'$ is the only unknown, we can find it by minimising $-D_{KL}$ between the conditional probability distributions:

12 $$\begin{aligned} z' = arg \min _{z'}(-D_{KL}(p_{x|z} || q_{x'|z'})) \end{aligned}$$

using the Nelder–Mead simplex algorithm^74^ and a start guess being the centroid of the embedding $X'$ of the nearest points *X*. If the start guess is not in the basin of attraction of the global minimum, it means that the new point is not like any points presented during training and the embedded point will shoot towards infinity^49^.

One instance of the segmentation algorithm was running for each of the two audio channels used in the experiment and a new feature vector was formed for each detected segment and placed in a queue. A pool of 6 workers (containers running in the cluster) processed feature vectors from the queue using the post-embedding algorithm described above and emitted events containing onset, bird ID, the low dimensional point, a letter representing the region it belonged to and the latency measured from the end of the segment until the event was emitted. The median latency over 3 million syllables was 1.089 s (percentiles: 5th = 0.356, 25th = 0.945, 75th = 1.304, 95th = 2.500). We found that 95% of the segments were classified and the remaining 5% were marked as unclassified.

Sequence events were generated, by event-processors in the cluster, based on the timing of the syllable events. If the onset of the next segment was within a window of 0.5 s after the offset of the previous, it was added to the sequence and otherwise it was assigned to a new sequence. Within 3 s after the end of a sequence an event was emitted containing onset, offset, bird id and the sequence.

To find the motif of the bird we formed a transition probability matrix based on the training data. Since the transitions in the motif were by far the most frequent, the syllables in the motif already stood out. Because the motif was repeated several times in a bout, they formed cycles in the transition matrix (Fig. 4H). We found the cycle by starting from the globally most frequent transition and following the locally most frequent transitions until getting back to an already visited element. If the motif contained repeated syllables or if the bird sang a lot of introductory notes, there was a possibility for dead ends, but they could be detected and solved programmatically.

The birds often sing variations of the long motif so we found the ten most common substrings of the motif and looked for those in the sequence events. We counted the number of occurrences of each substring in the sequence and if a motif was present, we emitted a motif event (based on the most frequent substring in the sequence) containing onset, motif, number of occurrences and bird id. The motif detector was implemented as an event-processor running in the cluster. To verify the motif detector, an observer (LBL) looked at the spectrograms of all the motifs generated during the 2 h of experimentation for one bird (96 motifs) and confirmed that all of them were indeed motifs.

## Data Availability

Code has been uploaded as a Jupyter notebook at: https://github.com/LeonBondeLarsen/tsne_classifier. The datasets generated during and/or analysed during the current study are available from the corresponding authors on reasonable request.

## References

[1] Patricelli GL, Coleman SW, Borgia G (2006). Male satin bowerbirds, ptilonorhynchus violaceus, adjust their display intensity in response to female startling: An experiment with robotic females. Anim. Behav..

[2] Reaney LT, Sims RA, Sims SW, Jennions MD, Backwell PR (2008). Experiments with robots explain synchronized courtship in fiddler crabs. Curr. Biol..

[3] Partan SR, Otovic P, Price VL, Brown SE (2011). Assessing display variability in wild brown anoles *Anolis sagrei* using a mechanical lizard model. Curr. Zool..

[4] Klein BA, Stein J, Taylor RC (2012). Robots in the service of animal behavior. Commun. Integr. Biol..

[5] Marras S, Porfiri M (2012). Fish and robots swimming together: Attraction towards the robot demands biomimetic locomotion. J. R. Soc. Interface.

[6] Polverino G, Phamduy P, Porfiri M (2013). Fish and robots swimming together in a water tunnel: Robot color and tail-beat frequency influence fish behavior. PLoS ONE.

[7] Kopman V, Laut J, Polverino G, Porfiri M (2013). Closed-loop control of zebrafish response using a bioinspired robotic-fish in a preference test. J. R. Soc. Interface.

[8] Bonnet F, Kato Y, Halloy J, Mondada F (2016). Infiltrating the zebrafish swarm: Design, implementation and experimental tests of a miniature robotic fish lure for fish-robot interaction studies. Artif. Life Robot..

[9] Partan SR, Fulmer AG, Gounard MA, Redmond JE (2010). Multimodal alarm behavior in urban and rural gray squirrels studied by means of observation and a mechanical robot. Curr. Zool..

[10] Benichov JI (2016). The forebrain song system mediates predictive call timing in female and male zebra finches. Curr. Biol..

[11] Michelsen A, Andersen BB, Storm J, Kirchner WH, Lindauer M (1992). How honeybees perceive communication dances, studied by means of a mechanical model. Behav. Ecol. Sociobiol..

[12] Halloy J (2007). Social integration of robots into groups of cockroaches to control self-organized choices. Science.

[13] de Margerie E, Lumineau S, Houdelier C, Yris MR (2011). Influence of a mobile robot on the spatial behaviour of quail chicks. Bioinspir. Biomimet..

[14] Romano D (2017). Multiple cues produced by a robotic fish modulate aggressive behaviour in siamese fighting fishes. Sci. Rep..

[15] Simon R (2019). Development and application of a robotic zebra finch (robofinch) to study multimodal cues in vocal communication. PeerJ.

[16] Dombeck DA, Reiser MB (2012). Real neuroscience in virtual worlds. Curr. Opin. Neurobiol..

[17] Steuer J (1992). Defining virtual reality: Dimensions determining telepresence. J. Commun..

[18] Ahrens MB (2012). Brain-wide neuronal dynamics during motor adaptation in zebrafish. Nature.

[19] Reiser MB, Dickinson MH (2008). A modular display system for insect behavioral neuroscience. J. Neurosci. Methods.

[20] Harvey CD, Collman F, Dombeck DA, Tank DW (2009). Intracellular dynamics of hippocampal place cells during virtual navigation. Nature.

[21] Kaupert U (2017). Spatial cognition in a virtual reality home-cage extension for freely moving rodents. J. Neurophysiol..

[22] Stowers JR (2017). Virtual reality for freely moving animals. Nat. Methods.

[23] Cong L (2017). Rapid whole brain imaging of neural activity in freely behaving larval zebrafish (*Danio rerio*). Elife.

[24] Rychen J (2021). A system for controlling vocal communication networks. Sci. Rep..

[25] Zann RA (1996). The Zebra Finch: A Synthesis of Field and Laboratory Studies.

[26] Ter Maat A, Trost L, Sagunsky H, Seltmann S, Gahr M (2014). Zebra finch mates use their forebrain song system in unlearned call communication. PLoS ONE.

[27] Anisimov VN (2014). Reconstruction of vocal interactions in a group of small songbirds. Nat. Methods.

[28] Elie JE, Theunissen FE (2016). The vocal repertoire of the domesticated zebra finch: A data-driven approach to decipher the information-bearing acoustic features of communication signals. Anim. Cogn..

[29] Davidson GL, Clayton NS (2016). New perspectives in gaze sensitivity research. Learn. Behav..

[30] Ljubičić I, Bruno JH, Tchernichovski O (2016). Social influences on song learning. Curr. Opin. Behav. Sci..

[31] Benichov JI, Vallentin D (2020). Inhibition within a premotor circuit controls the timing of vocal turn-taking in zebra finches. Nat. Commun..

[32] Galoch Z, Bischof H-J (2006). Zebra finches actively choose between live images of conspecifics. Ornithol. Sci..

[33] Galoch Z, Bischof H-J (2007). Behavioural responses to video playbacks by zebra finch males. Behav. Proc..

[34] Adret P (1997). Discrimination of video images by zebra finches (*Taeniopygia guttata*): Direct evidence from song performance. J. Comp. Psychol..

[35] Ikebuchi M, Okanoya K (1999). Male zebra finches and bengalese finches emit directed songs to the video images of conspecific females projected onto a tft display. Zoolog. Sci..

[36] Chen Y, Matheson LE, Sakata JT (2016). Mechanisms underlying the social enhancement of vocal learning in songbirds. Proc. Natl. Acad. Sci..

[37] Carouso-Peck S, Goldstein MH (2019). Female social feedback reveals non-imitative mechanisms of vocal learning in zebra finches. Curr. Biol..

[38] Sober SJ, Brainard MS (2009). Adult birdsong is actively maintained by error correction. Nat. Neurosci..

[39] Hoffmann LA, Kelly CW, Nicholson DA, Sober SJ (2012). A lightweight, headphones-based system for manipulating auditory feedback in songbirds. J. Vis. Exp. JoVE.

[40] Larsen LB, Neerup MM, Hallam J (2021). Online computational ethology based on modern it infrastructure. Eco Inform..

[41] Cugola G, Margara A (2012). Processing flows of information: From data stream to complex event processing. ACM Comput. Surv. CSUR.

[42] Elie JE (2010). Vocal communication at the nest between mates in wild zebra finches: A private vocal duet?. Anim. Behav..

[43] Sossinka R, Böhner J (1980). Song types in the zebra finch *Poephila guttata* Castanotis 1. Z. Tierpsychol..

[44] Jarvis ED, Scharff C, Grossman MR, Ramos JA, Nottebohm F (1998). For whom the bird sings: Context-dependent gene expression. Neuron.

[45] Kao MH, Doupe AJ, Brainard MS (2005). Contributions of an avian basal ganglia-forebrain circuit to real-time modulation of song. Nature.

[46] So LY, Munger SJ, Miller JE (2019). Social context-dependent singing alters molecular markers of dopaminergic and glutamatergic signaling in finch basal ganglia area x. Behav. Brain Res..

[47] Maaten Lvd, Hinton G (2008). Visualizing data using t-SNE. J. Mach. Learn. Res..

[48] Meyer F (1994). Topographic distance and watershed lines. Signal Process..

[49] Berman GJ, Choi DM, Bialek W, Shaevitz JW (2014). Mapping the stereotyped behaviour of freely moving fruit flies. J. R. Soc. Interface.

[50] Tchernichovski O, Nottebohm F, Ho CE, Pesaran B, Mitra PP (2000). A procedure for an automated measurement of song similarity. Anim. Behav..

[51] Kollmorgen S, Hahnloser RH, Mante V (2020). Nearest neighbours reveal fast and slow components of motor learning. Nature.

[52] Grosenick L, Marshel JH, Deisseroth K (2015). Closed-loop and activity-guided optogenetic control. Neuron.

[53] Nourizonoz A (2020). EthoLoop: Automated closed-loop neuroethology in naturalistic environments. Nat. Methods.

[54] Lohr B, Wright TF, Dooling RJ (2003). Detection and discrimination of natural calls in masking noise by birds: Estimating the active space of a signal. Anim. Behav..

[55] Brumm H, Slabbekoorn H (2005). Acoustic communication in noise. Adv. Study Behav..

[56] Riedner, D. & Adam, I. Units of motor production: Bengalese finches interrupt song within syllables. bioRxiv (2020).

[57] Emmerton J (1983). Pattern discrimination in the near-ultraviolet by pigeons. Percept. Psychophys..

[58] Nuboer J, Coemans M, Vos J (1992). Artificial lighting in poultry houses: Do hens perceive the modulation of fluorescent lamps as flicker?. Br. Poult. Sci..

[59] Jakobsen L, Christensen-Dalsgaard J, Juhl PM, Elemans CP (2021). How loud can you go? physical and physiological constraints to producing high sound pressures in animal vocalizations. Front. Ecol. Evol..

[60] Brumm H, Slater PJ (2006). Animals can vary signal amplitude with receiver distance: Evidence from zebra finch song. Anim. Behav..

[61] Andreassen T, Surlykke A, Hallam J (2014). Semi-automatic long-term acoustic surveying: A case study with bats. Eco. Inform..

[62] Younkin AC, Corriveau PJ (2008). Determining the amount of audio-video synchronization errors perceptible to the average end-user. IEEE Trans. Broadcast..

[63] Martin, J., Burbank, J., Kasch, W. & Mills, P. D. L. Network Time Protocol Version 4: Protocol and Algorithms Specification. RFC 5905. 10.17487/RFC5905 (2010).

[64] Lichvar, M. C. https://chrony.tuxfamily.org/, accessed 9 December 2020 (1999).

[65] Birman K, Joseph T (1987). Exploiting virtual synchrony in distributed systems. ACM SIGOPS Oper. Syst. Rev..

[66] Weil, S. A., Brandt, S. A., Miller, E. L., Long, D. D. & Maltzahn, C. Ceph: A scalable, high-performance distributed file system. In *Proceedings of the 7th symposium on Operating systems design and implementation*, 307–320 (2006).

[67] Merkel D (2014). Docker: lightweight linux containers for consistent development and deployment. Linux J..

[68] Gstreamer. *GStreamer*. https://gstreamer.freedesktop.org/, accessed 28 August 2018 (2001).

[69] Vohra, D. Apache Kafka. In *Pract. Hadoop Ecosyst*, 339–347, 10.1007/978-1-4842-2199-0_9 (Apress, Berkeley, CA, 2016).

[70] Schmitt OH (1938). A thermionic trigger. J. Sci. Instrum..

[71] Breiman L (2001). Random forests. Mach. Learn..

[72] De Cheveigné A, Kawahara H (2002). YIN, a fundamental frequency estimator for speech and music. J. Acoust. Soc. Am..

[73] Omohundro SM (1989). Five Balltree Construction Algorithms.

[74] Nelder JA, Mead R (1965). A simplex method for function minimization. Comput. J..

